# Personalized Oral and Dental Care

**DOI:** 10.3390/jpm13010110

**Published:** 2023-01-04

**Authors:** Alessandra Amato

**Affiliations:** Department of Neuroscience, Reproductive Science and Dentistry, University of Naples Federico II, 80131 Naples, Italy; aale.amato@gmail.com

Recent advances in genomics, data analytics technologies, and biotechnology have been unprecedented, ushering in a new era of healthcare in which interventions are increasingly tailored to individual patients. 

Unlike traditional one-size-fits-all strategies, this precision approach to healthcare allows individuals to receive treatments based on their own characteristics, which are entirely unique from a genetic, biological, and social perspective. 

Approaches based on precision medicine can be extended to oral health, which is an essential component of general health, allowing for a deeper integration of oral and systemic healthcare [1]. 

The personalization of oral and dental care requires a shift in focus to the individual patient, considered as a whole, to ensure that precision prevention, treatment, and public health interventions are available to the population but based on specific and individual genetic, biological, behavioral, and social determinants [2].

With these goals, translational research is tasked with gaining a more comprehensive understanding of the factors underlying oral and general healthy conditions and those that may contribute to the development of oral and systemic diseases and disorders, analyzing the human genome, epigenome, proteome, microbiome, and other complex biological systems [3].

Progresses in the use of saliva in diagnostics, has enabled the detection of both dental and systemic diseases through potential indicators of heart disease, post-traumatic stress disorder, oral and non-oral cancers, immunodeficiencies, viruses, and others [4,5,6,7,8]. 

Advances in nanotechnology will enable the development of implantable biodevices in the oral cavity that can be used for both programmed and individualized drug delivery and oral health monitoring [9,10,11,12]. Such technology, coupled with a deeper understanding of the genes and mechanisms involved in orofacial and dental pain and assisted by clinical decision support tools that can process information from the patient’s dental record, may aid in personalized oral and systemic pain management [13,14,15].

Oral oncology may benefit from personalized approaches, especially oral squamous cell carcinoma diagnosis and treatment. Investigations based on biomarker detection would make it possible to diagnose these types of carcinomas at an early stage and stop their development. Moreover, once the type of tumor is ascertained, the oncologist or oral pathologist could determine which therapy would be best tailored to the patient’s genetic characteristics, increasing the chances of treatment success [16].

Personalized dentistry could be applied to early childhood caries, which are the most common chronic disease in childhood. Indeed, their onset is undoubtedly related to environmental aspects, such as fluoride intake and dietary habits, as well as individual aspects, such as dental anatomy, enamel quality, microbiome and salivary composition, and immune defenses, but is currently thought to be linked to genetics in about 40–70% of disease occurrence [17].

Similarly, personalized dentistry may also have applications in orthodontics, as the etiology of malocclusions is highly dependent on both genetic and environmental causes [4,18].

Genomics will also play an increasingly important role in preventing and treating periodontitis [19,20], a polymicrobial infection whose clinical variability depends on genetic factors [21,22,23]. Some people are actually more susceptible to periodontitis and peri-implantitis, as well as to multiple interconnected systemic infective, inflammatory, and neoplastic diseases, than others [9,20,24].

The potential personalized oral and dental care interventions described should, therefore, emphasize disease prevention through individualized rather than population-based surveillance, improve diagnosis through more accurate genetic testing, promote earlier detection of abnormalities and avoid further invasive and expensive testing, reduce errors and side effects, and select appropriate medications in more precise and specific doses when prescribing medications [15].

Although the body of evidence is growing, much remains to be done to translate it into clinical practice [18]. For the moment, personalized dentistry is primarily a fertile field of research, but clinical applications are just around the corner.

## Data Availability

Data supporting reported results are available on PubMed/MEDLINE, Scopus, and Web of Science databases.
